# Metabolomic insights into the browning of the peel of bagging ‘Rui Xue’ apple fruit

**DOI:** 10.1186/s12870-021-02974-y

**Published:** 2021-05-08

**Authors:** Hui Wang, Shuang Wang, Miao-Miao Fan, Shu-Hui Zhang, Lu-Long Sun, Zheng-Yang Zhao

**Affiliations:** 1 College of Horticulture, Northwest A & F University, Yangling, Xianyang, 712100 Shaanxi China; 2 College of Horticultural Science and Engineering, Shandong Agricultural University / State Key Laboratory of Crop Biology, Taian, 271018 Shandong China

**Keywords:** *Malus domestica* Borkh., Fruit bagging, ‘Rui Xue’, Browning, Metabolomic

## Abstract

**Background:**

Bagging is one of the most important techniques for producting high-quality fruits. In the actual of cultivating, we found a new kind of browning in peel of apple fruit that occurs before harvest and worsen during storage period. There are many studies on metabonomic analysis of browning about storage fruits, but few studies on the mechanism of browning before harvest.

**Results:**

In this study, five-year-old trees of ‘Rui Xue’ (CNA20151469.1) were used as materials. Bagging fruits without browning (BFW) and bagging fruits with browning (BFB) were set as the experimental groups, non-bagging fruits (NBF) were set as control. After partial least squares discriminant analysis (PLS-DA), 50 kinds of metabolites were important with predictive VIP > 1 and *p*-value < 0.05. The most important differential metabolites include flavonoids and lipids molecules, 11 flavonoids and 6 lipids molecules were significantly decreased in the BFW compared with NBF. After browning, 11 flavonoids and 7 lipids were further decreased in BFB compared with BFW. Meanwhile, the significantly enriched metabolic pathways include galactose metabolism, ABC membrane transporter protein, flavonoid biosynthesis and linoleic acid metabolism pathways et al. Physiological indicators show that, compared with NBF, the content of malondialdehyde (MDA), hydrogen peroxide (H_2_O_2_)_,_ superoxide anion (O_2_^−^) in peel of BFW and BFB were significantly increased, and the difference of BFB was more significant. Meanwhile, the antioxidant enzyme activities of BFW and BFB were inhibited, which accelerated the destruction of cell structure. In addition, the metabolome and physiological data showed that the significantly decrease of flavonoid was positively correlated with peel browning. So, we analyzed the expression of flavonoid related genes and found that, compared with NBF, the flavonoid synthesis genes *MdLAR* and *MdANR* were significantly up-regulated in BFW and BFB, but, the downstream flavonoids-related polymeric genes *MdLAC7* and *MdLAC14* were also significantly expressed.

**Conclusions:**

Our findings demonstrated that the microenvironment of fruit was changed by bagging, the destruction of cell structure, the decrease of flavonoids and the increase of triterpenoids were the main reasons for the browning of peel.

**Supplementary Information:**

The online version contains supplementary material available at 10.1186/s12870-021-02974-y.

## Background

Apple is one of the most produced fruits in the world, which occupies an important position in the world fruit trade [1]. China has become the largest producer of apple in the world (Food and Agriculture Organization of the United Nations, FAO). In 2018, China’s apple cultivation area has reached 2.072 million hm^2^, accounting for 57.7% of the world’s total cultivation area, and the total production reached 39.235 million tons, accounting for 54.5% of the world’s total apple production.

The fruit with bright color, smooth surface and no pesticide residue is favored by consumers at present. Fruit bagging as one of the most important good agricultural practice to produce high quality fruit [2, 3]. It has been used in several fruit crops to improve the peel color and surface smoothness, and to reduce the incidence of disease, insect pests, sunburn of the peel, and bird damage [3–8]. It has been widely used in China, Australia, Japan, and the United States for the cultivation of peach, apple, pear, grape, loquat and so on [3, 9]. However, bagging can also induce some negative effects on fruits. The microenvironment after bagging will reduce the thickness of peel and the content of sugar, acid, mineral elements, and other internal soluble substances in fruits [7, 10]. After bagging, the dark condition could inhibit the synthesis and accumulation of antioxidant substances, such as flavonoids and phenols in fruits [11–13], thereby reduced the fruits’ resistance to stress.

**Fig. 1:**
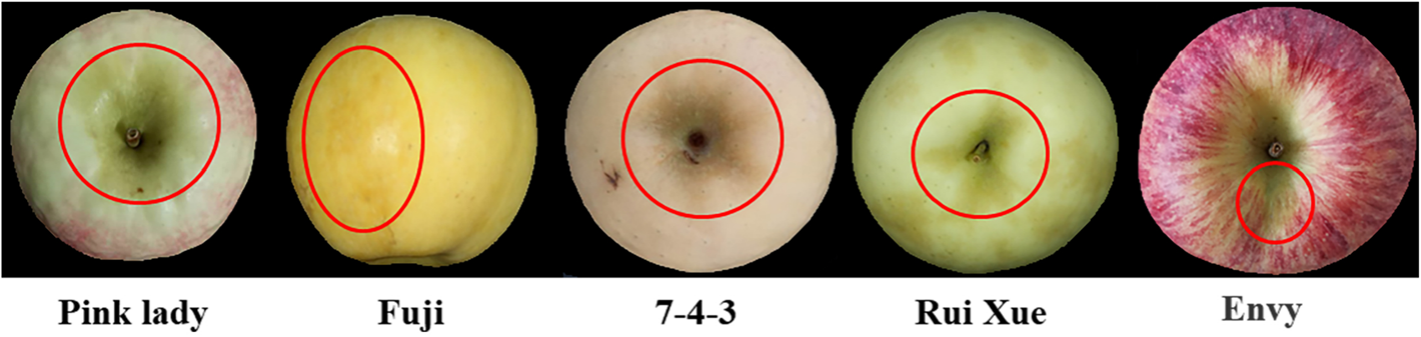
Investigation on different late-ripening varieties about peel browning

**Fig. 2:**
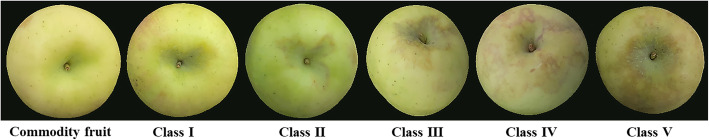
The classification in apple peel browning about bagging ‘Rui Xue’

One interesting phenomenon was observed that browning in peel was easily occurred in bagging fruits, especially some late-ripening varieties, including ‘Fuji’, ‘Golden Delicious’, ‘Pink Lady’, ‘Envy’, ‘Rui Xue’, and so on, which occurred at the late stage of fruit growth (from September to October) in the field (Fig. 1). While no browning occurred in unbagging fruits. The new kind of browning is different from the superficial scald, which occurs before harvest and worsen during storage period, and flavonoids were significantly reduced and triterpenoids increased along with browning. While, superficial scald only occurs during storage, and α-farnesene synthase (*MdAFS1*) of TPS enzymes and its oxidative product have been identified as key factors causing superficial scald [14]. Before harvest, browning started at stalk cavity and then expanded to the whole surface. Meanwhile, the stalk cavity and other expanded part changes from pale brown to brown and dark brown (Fig. 2). During storage, the healthy surface deteriorates gradually by outward diffusion from the stalk cavity. Seriously, the brown part could penetrate into the fruit interior, without any peculiar smell. Although the new kind of browning doesn’t affect its taste, the appearance of fruit was influenced, which reduces its value [15, 16].

Previous studies on fruit browning mainly focused on storage period. Including long-term low temperature-stored induced the peel browning of pears, apples, mangoes, bananas and so on [17–19]. Luo discovered that the antioxidant and redox system are directly linked to apple soft scald development at low temperature by metabonomics techniques [19]. Jordi indicated the mechanism of ethylene mediated pear peel browning under low temperature was explained by metabonomic techniques [20]. Chen also used metabonomic techniques suggested that peel browning could induce the conversion of metabolic products from amino acids to terpenes [17]. Previous analyses have revealed changes in the metabolism of reactive oxygen species generated by the tricarboxylic acid cycle (TCA) and γ-aminobutyric acid (GABA) shunt, which causes stressed conditions inside the mesocarp. Further, reduced levels of antioxidants and enzymes dissipating reactive oxygen species in mesocarp deteriorate the fruit physiology. This oxidative stress all along aects the level of amino acids, sugars and enzymes responsible for flavor generation in the fruit [18, 19, 21]. However, how bagging induced browning has not been thoroughly studied. In addition, few studies have attempted to use comparative metabolomics to investigate the mechanism of peel browning before harvest.

We hypothesized that bagging changed the microenvironment of apple fruit, reducing the antioxidant capacity of peel, leading to a thinner peel, and in the regions with large differences in temperature between day and night, the antioxidant content in fruit is reduced, the structure of the cells is altered, and enzymatic browning of the fruit on the tree is induced after the cell membrane broken. Here, we conducted a metabolomic analysis to study the browning mechanism of apple peel using the sensitive variety ‘Rui Xue’. The findings were helpful for our understanding in the physiological changes of peel before harvest, refining the cultivation techniques before harvest and storage techniques.

## Results

### The effects of bagging on peel thickness

**Fig. 3:**
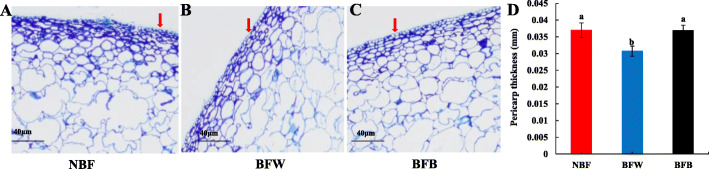
The effect of bagging on peel thickness. Note: **a**: Unbagging fruit; **b**: Bagging fruit without browning; **c**: Bagging fruit with browning; **d**: Thickness of apple peel

The peel plays an important role in protecting the fruit from outside stimulations [22, 23]. We measured the thickness of the peel to determine whether bagging alters the cellular structures. The epithelial cells of the NBF included 5–6 layers (Fig. 3a), while the upper epidermal cells of the bagging fruit (BFW and BFB) had only 4–5 layers (Fig. 3b-c), indicating that bagging changed the microenvironment around the fruit and reduced the number of cell layers in the peel.

The peel thickness of the BFW was only 0.031 mm, which was significantly reduced (by 16.2%) compared with NBF (Fig. 3d). However, the peel thickness of the BFB did not decreased, but increased significantly (by 16.0%) compared with the BFW, indicating that the cell walls of the peel were thickened.

### The effects of bagging on the microstructure of peel cells

**Fig. 4:**
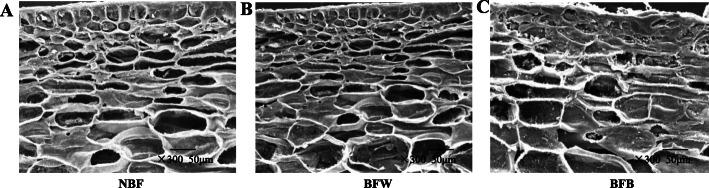
The effects of bagging on the microstructure of peel cells. **a**: Bagging fruit without browning; **b**: Bagging fruit with browning

We used transmission electron microscopy to determine the microstructure of the peel. The NBF and BFW fruits maintained the original tissue structure, with a uniform, complete and clear honeycomb structure, smooth and flat cell surface (Fig. 4a-b). The cellular structure of the BFB was obviously deformed and shriveled, the subcutaneous cells had begun to sag, the structure was loose and fluffy, the cellular structure was no longer honeycombed and complete, the surface tissue was broken, and the cellular structure was destroyed (Fig. 4c).

### Metabolomic analysis

#### Principal component analysis and metabolite intensity distribution assessment of QC samples

**Fig. 5:**
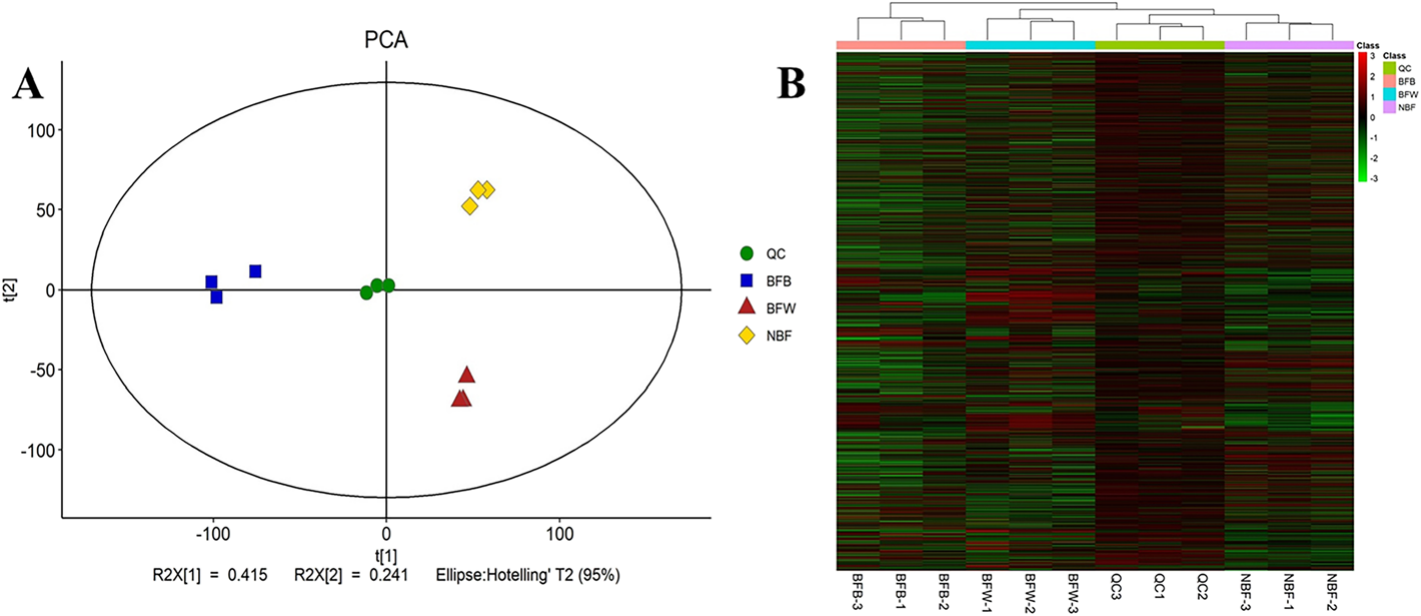
Principal component analysis of QC samples and analysis of metabolite strength distribution. Note: **a**: PCA model figure; **b**: Hierarchical clustering Fig. QC: Quality control; BFB: Bagging fruit with browning; BFW: Bagging fruit without browning; NBF: Unbagging fruit

To test the stability of the mass spectrometry system, we mixed (in equal proportions) samples to create QC samples. Firstly, we analyzed the principal components of QC samples; the PCA model diagram obtained through 7-fold cross-validation (seven cycles of cross-validation) is shown in Fig. 5a. The QC samples were closely clustered together, indicating that the experiment was stable and repeatable. Hierarchical clustering for the expression levels of all metabolites intuitively demonstrated the stability of the relationship between QC and other samples (Fig. 5b). There was no significant differences in the intensity distribution of metabolites between the QC and the other three samples, indicating a stable and accurate data.

#### Determination of metabolites from the browning part of the peel

**Fig. 6:**
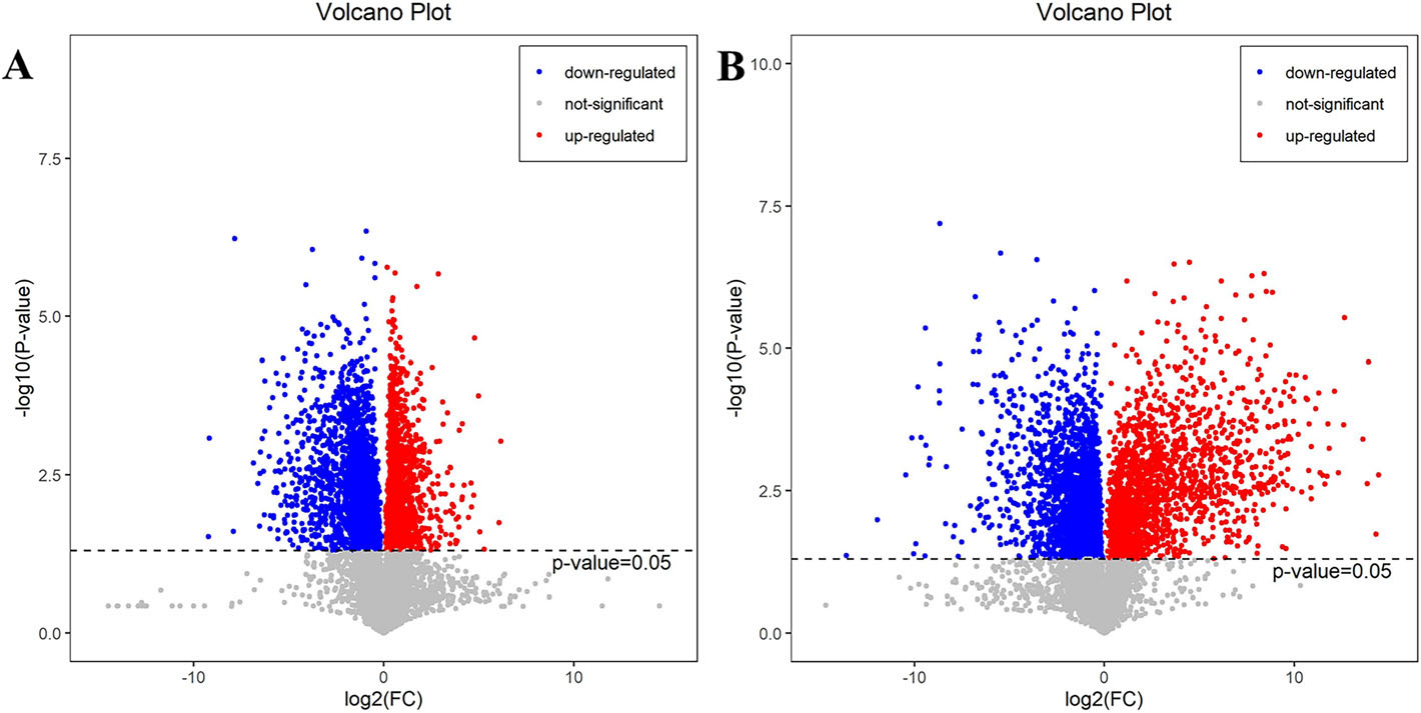
The effect of fruit peel browning on metabolites. **a**: BFW-NBF group; **b**: BFW-BFB group. Note: The red origin represents the significantly up-regulated differential metabolites in the experimental group, the blue origin represents the significantly down-regulated differential metabolites, and the gray point represents no significant difference

We analyzed the differences of all metabolites in NBF, BFW, and BFB. Compared with NBF, 1775 and 2089 metabolites were significantly up-regulated and down-regulated in BFW, respectively (Fig. 6a). Compared with BFW, 2211 and 2534 metabolites were significantly up-regulated and down-regulated in BFB (Fig. 6b). To screen the marker metabolites, a combination of multi-dimensional and single-dimensional analysis was used. Differential metabolites with VIP > 1 and *p*-value < 0.05 were regarded as significantly differential metabolites. One hundred thirty-one metabolites of BFW were significantly up-regulation and 217 metabolites down-regulation compared with NBF; 198 metabolites of BFB were significantly up-regulationand and 178 metabolites down-regulation compared with BFW.

#### Screening of differential metabolites

To investigate the relationship among samples and the differences in metabolites, a hierarchical clustering analysis was conducted on the expression levels of all those metabolites where a significant difference had been detected. According to the VIP value, a visual analysis was performed for the expression levels of the first top 50 differential metabolites. The results showed that, compared with NBF, 13 flavonoids (including apigenin, rutin, myricetin 3-glucoside, isoquercitrin, quercetin, quercetin 3-o-glucoside, phloridzin, 6-hydroxyluteolin 6-xyloside, procyanidin B2, quercetin 3-arabinoside and morin were significantly decreased, clausarinol and gingerenone B were significantly increased), ten carbohydrate compounds (including lactulose, *d*-mannitol, sucrose, *d*-galactose, *d*-maltose, maltotriose, glucose 1-phosphate, fructofuranosy and 3-fucosyllactose were significantly increased, *d*-glucopyranoside was significantly decreased), ten lipid molecules (*d*-glucopyranoside, hydroxypregn sulfate, 1-hexanol arabinosylglucoside, corchoionol C 9-glucoside and vomifoliol were significantly decreased, 2-hydroxyadipic acid, SM(d18:1/18:1(9Z)), scillirosidin, PI (15:0/0:0) and C16 sphinganine were significantly increased), nine benzene ring-type compounds (including eugenol, anthraquinone and buclizine were significantly decreased, benzene-1,3,5-triol, thiabendazole, triphenyl-phosphate-thiabendazole, pandamarilactam-3x, citbismine C and pterosin H were significantly increased), five organic acids and their derivatives (including raltitrexed, isocitrate and 1-o-p-coumaroyl-beta-d-glucose were significantly increased, ustiloxin D and oxane-2-carboxylic acid were significantly decreased), one scatterbrained lipid (armillaripin was significantly increased) and two other types metabolites (including 2,2-dichloro-1,1-ethanediol was significantly increased, atorvastatin was significantly decreased) had significantly changed in BFW (Fig. S1A, Table S1).

The differential metabolites were further subdivided. The down-regulated differential metabolites were mainly flavonoids in BFW compared with NBF. It is well known that most plants contain flavonoids [24], which play an important role in plant growth. As a kind of secondary metabolite, flavonoids can remove free radicals and delay the normal tissue cell apoptosis [25]. However, flavonoids were not included among the up-regulated metabolites in BFW. To determine the causes of browning, or the substances produced by browning, the metabolites were detected in BFB and BFW groups. Among the significantly changed metabolites (Fig. S1B), we identified 12 flavonoids (including procyanidin-B2, phloridzin, hydroxyluteolin, quercetin 3-arabinoside, gingerenone B, quercetin-3-o-glucoside, isoquercitrin, quercitrin, morin and clausarinol were significantly decreased, eurycomanone and marmesin galactoside were significantly increased), 13 triterpenes (including ganosporeric acid A was significantly decreased, medicagenic acid, 12-oleanadien-28-oic acid, esculentic acid, oxane-2-carboxylic acid, phytolaccinic acid, ganolucidic acid B, protobassic acid, ganolucidic acid E, 16alpha-hydroxygypsogenic acidid, pokeberrygenin and corosin were significantly increased), 11 lipids and their associated molecules (including SM(d18:1/18:1(9Z)), C16-sphinganine, scillirosidin, estradiol-17beta-3-sulfate, 2-hydroxyadipic acid and vomifoliol, tetranor-PGDM were significantly decreased, physalolactone B, EB-1213, pentanorcholecalciferol and cyclo-cholest were significantly increased), 7 organic acids and their derivatives (including chlorogenic acid, feruloyl C1-glucuronide, cis-5-caffeoylquinic acid, raltitrexed and quinic acid were significantly decreased, acety-l-tributy-l-citrate and isocitrate were significantly increased,), 4 sugar compounds (including *d*-maltose, *d*-Fructofuranosyl, 3-fucosyllactose and sucrose were significantly decreased), 2 benzene ring-type compound derivatives (including citbismine C was significantly decreased, C2-(4-Methyl-1,3-pentadienyl) anthraquinone was significantly increased), 1 lipid (armillaripin was significantly decreased), and 1 other compound ((17Z)-1α,25-dihydroxy-26,27-dimethyl-17,20,22,22,23,23-hexadehydrocholecalciferol was significantly increased) had significantly changed in BFB (Fig. S1B, Table S2).

#### Enrichment analysis of differential metabolites

**Fig. 7:**
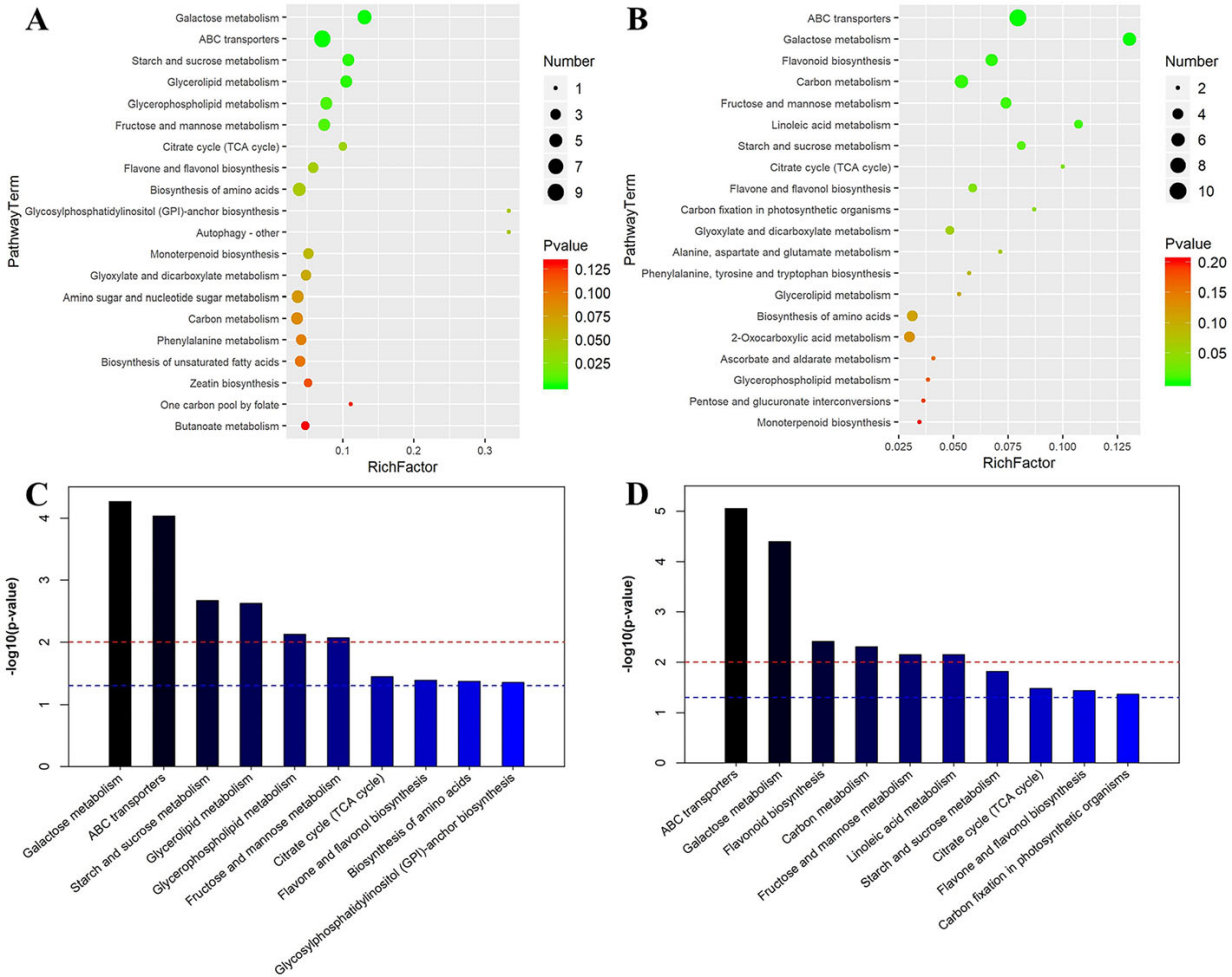
Enrichment analysis of metabolic pathways of different metabolites. **a,c**: BFW-NBF group; **b,d**: BFW-BFB group. Note: The ordinate is the name of metabolic pathway; the abscissa is the enrichment factor (Rich factor = the number of metabolites with significant difference/the number of total metabolites in the pathway). The color from red to green means that the *p*-value decreases successively

To understand the differential metabolic pathways of various samples by comparing the differential metabolites in the Kyoto Encyclopedia of Genes and Genomes (KEGG, https://www.kegg.jp/) database, the top 20 metabolic pathways with significant enrichment were selected for bubble mapping (Fig. 7). Galactose metabolism, ABC membrane transporter protein, starch and sucrose metabolism, glyceride/phospholipid metabolism, and fructose and mannose metabolism were significantly changed in BFW compared with NBF (Fig. 7a-c).

To better understand the final major metabolites and metabolic pathways of bagging-induced fruit browning, we conducted enrichment analysis of differential metabolites and metabolic pathways in BFW and BFB. Multiple metabolic pathways were significantly changed in BFB compared with BFW: ABC membrane transporter, galactose metabolism, flavonoid biosynthesis, carbon metabolism, fructose and mannose metabolism, and linoleic acid metabolism (Fig. 7b-d). Based on the top 50 differential metabolites, the inhibition of flavonoid biosynthesis leads to the decrease of antioxidant substances, which is directly responsible for fruit browning, while the damage of the cell membrane might be the direct cause of the inhibition of flavonoid biosynthesis and transport.

#### The effects of bagging on membrane lipid peroxidation

**Fig. 8:**
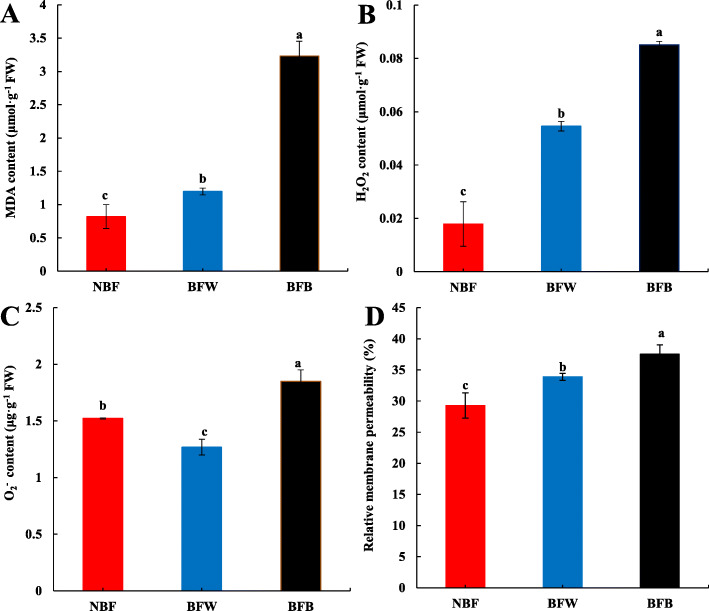
The relationship between membrane lipid peroxidation and peel browning. **a**: Malondialdehyde content (MDA); **b**: Relative membrane permeability; **c**: Hydrogen peroxide content (H_2_O_2_); **d**: Superoxide anion content (O_2_^−^).Error bars represent the averages of three biological replicates ± SD. Different letters represent differences between different processes. (Significance was defined as *P* < 0.05), same as the below

By measuring the microstructure of the peel, we determined that the browning after bagging was closely related to the integrity of the cell structure. We further measured the degree of membrane lipid peroxidation. Compared with NBF, the MDA content in peel of BFW and BFB were significantly increased by 0.46 times and 2.94 times, respectively (Fig. 8a); the H_2_O_2_ content in BFW and BFB were significantly increased by 2.05 times and 3.75 times, respectively (Fig. 8b); and the O_2_^−^ content was significantly decreased 16.6% in BFW, but significantly increased 21.5% in BFB (Fig. 8c). This finding indicated that the peroxidation of the cell membrane was accelerated by the microenvironment changes after bagging. We also measured the cell membrane permeability and found that, compared with NBF, the cell membrane permeability of BFW and BFB were significantly increased by 15.7 and 28.2%, respectively (Fig. 8d).

#### The effects of bagging on the activities of SOD, CAT and POD

**Fig. 9:**
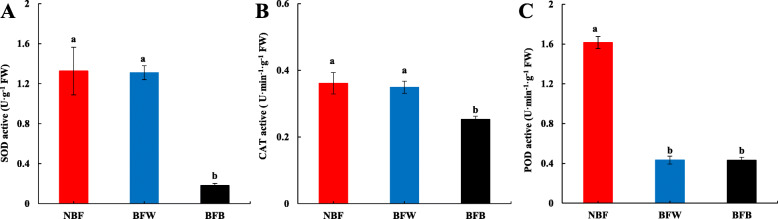
The effects of bagging on the activities of SOD, CAT and POD. **a**: Superoxide dismutase (SOD); **b**: Catalase from micrococcus lysodeiktic (CAT); **c**: Peroxidase (POD)

SOD, CAT and POD, the key active oxygen-scavenging enzymes, can scavenge ROS and prevent the accumulation of ROS [26–28]. As shown in Fig. 9, the activities of SOD, CAT and POD were significantly influenced after bagging. Compared with NBF, the activities of SOD and CAT in BFB were significantly decreased 82.3 and 30.1%, respectively, there were a downward trend, but no significances in BFW (Fig. 9a-b). Besides, the activities of POD was significantly decreased after bagging, compared with NBF, the activity of POD in peel of BFW and BFB were significantly decreased 73.2 and 73.3%, respectively (Fig. 9c).

#### The effects of bagging on the antioxidant capacity of the peel

**Fig. 10:**
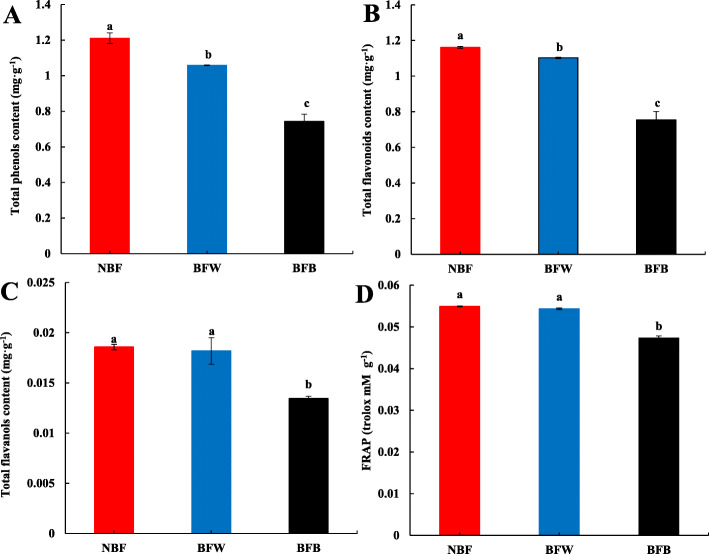
Relationship between antioxidant capacity and browning of peel

Based on metabolomics analysis, bagging significantly reduced the accumulation of antioxidant substances in the peel, inhibited the synthesis of flavonoids after browning. To further verify whether the decrease in antioxidant leading to the browning, we measured the content of total phenols (TP), total flavanols (TFA), and total flavonoids (TFO) in the peel. As shown in Fig. 10, after bagging, the accumulation of antioxidant substances was significantly reduced, and the reduction degree in BFB was more significant. Compared with NBF, the contents of TP and TFO were significantly reduced 12.6 and 5.1% in BFW, respectively, and the content of TFA showed a decreasing but not significant trend. Compared with NBF, the contents of TP, TFO, and TFA in the BFB were significantly reduced 38.6, 35.1 and 28.0%, respectively.

#### The effects of bagging on the phenolic acid composition of the peel

**Fig. 11:**
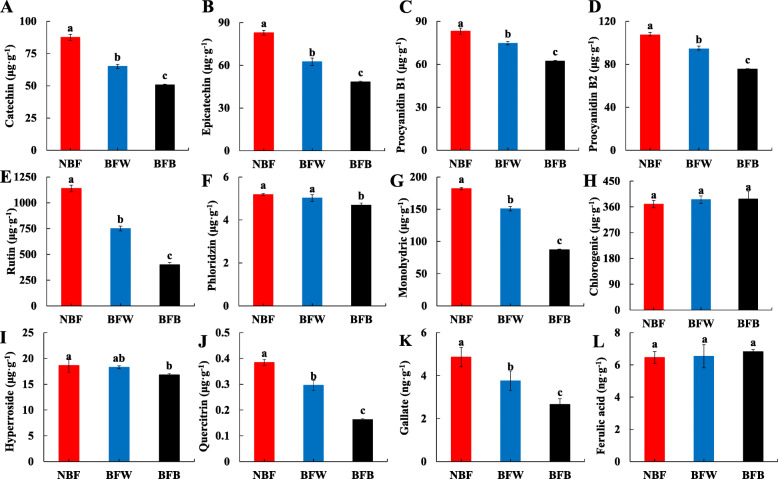
The effects of bagging on the phenolic acid composition of the peel

Phenolics acid in fruits and vegetables have gained much attention because of their antioxidant activities and their beneficial implications for human health [29]. By measuring the phenolic acid composition in the peel, we found that, after bagging, the phenolic acid in the peel was significantly reduced. As shown in the Fig. 11, compared with NBF, especially, the contents of catechin, epicatechin, procyanidin B1, procyanidin B2, rutin, monohydric acid, quercitrin, gallate in BFW were significantly reduced 25.8, 24.6, 10.1, 12.1, 34.1, 17.2, 21.1, 22.6%, respectively; after the peel browning, these substances content in BFB were significantly decreased 42.1, 41.6, 25.1, 29.7, 65.0, 52.1, 57.9, 45.3%, respectively. It further explained that, bagging affected the accumulation of antioxidant in peel.

#### Analysis the gene expression of related-flavonoids

**Fig. 12:**
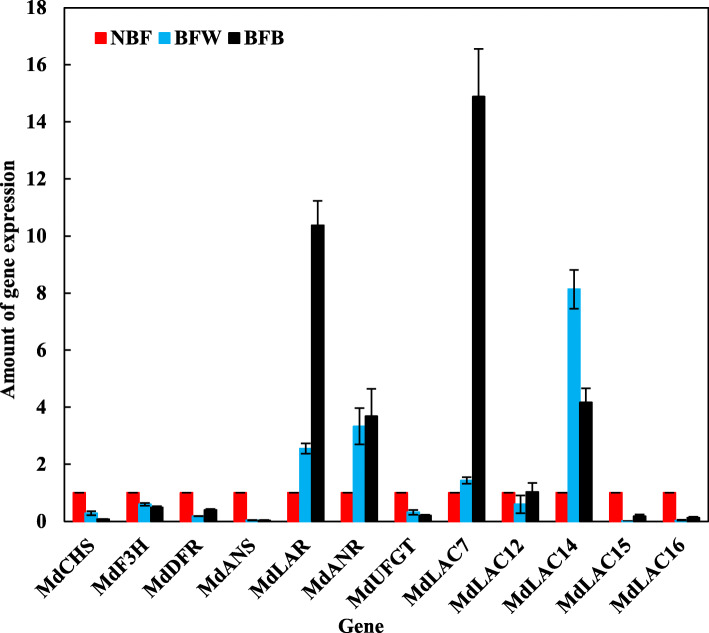
Analysis the gene expression of related-flavonoids

As shown in Fig. 12, compared with NBF, the flavonoid synthesis genes *MdCHS*, *MdF3H*, *MdDFR*, *MdANS* and *MdUFGT* were significantly down-regulated in BFW and BFB, while the *MdLAR* genes were significantly up-regulated by 2.55 and 9.37 times in BFW and BFB, and the *MdANR* genes were significantly up-regulated by 2.33 and 2.69 times in BFW and BFB, respectively. Interestingly, with the high expression of flavonoids-related synthetic genes, the downstream flavonoids-related polymeric genes were also highly expressed, among which *MdLAC7* and *MdLAC14* were the most significantly expressed. Compared with NBF, *MdLAC7* was significantly up-regulated by 0.43 and 13.88 times in BFW and BFB, and *MdLAC14* was significantly up-regulated by 7.13 and 3.17 times in BFW and BFB, respectively.

## Discussion

Browning in fruit, including enzymatic and non-enzymatic, the occurrence of enzymatic browning is closely related to the integrity of cell membrane. Enzymatic browning is induced by enzymes distributed in the elastoplast and substrates [30]. When cells suffered from stress, the ability of the fruit to clear free radicals was decreased, which lead to the accumulation of reactive oxygen species (O_2_^−^), accelerated membrane lipid peroxidation, and increased membrane permeability [30]. This conclusion is consistent with the results we have measured (Fig. 8), after bagging, more H_2_O_2_, O_2_^−^ and MDA were produced in the peel, which accelerated the destruction of cell structure. In addition, previous studies have found that the oxidation of chlorogenic acid by the enzyme polyphenol oxidase (PPO) is the key factor in the symptoms of the peel browning [31, 32]. During storage at low temperature, the cellular organelle’ internal membranes was destroyed, promoting the reaction between PPO (stored in the plastid) and chlorogenic acid (stored in the vacuole), leading to the production of quinones and melanin, which finally resulting in the dark-discolored areas on the fruit peel [20, 33]. Wang studied that, such as in the no browning peel of apple, its high expression of chlorogenic acid content, may be associated with inhibition of peel browning, exogenous chlorogenic acid treatment significantly enhanced the expression of genes involved in the biosynthesis of flavonoids, lignin synthesis gene was inhibited expression [34]. But, our results showed that endogenous chlorogenic acid content did not reduce with browning (Fig. 11), so, we thought that chlorogenic acid was not the most suitable substrate for enzymatic browning in before-harvest.

**Fig. 13:**
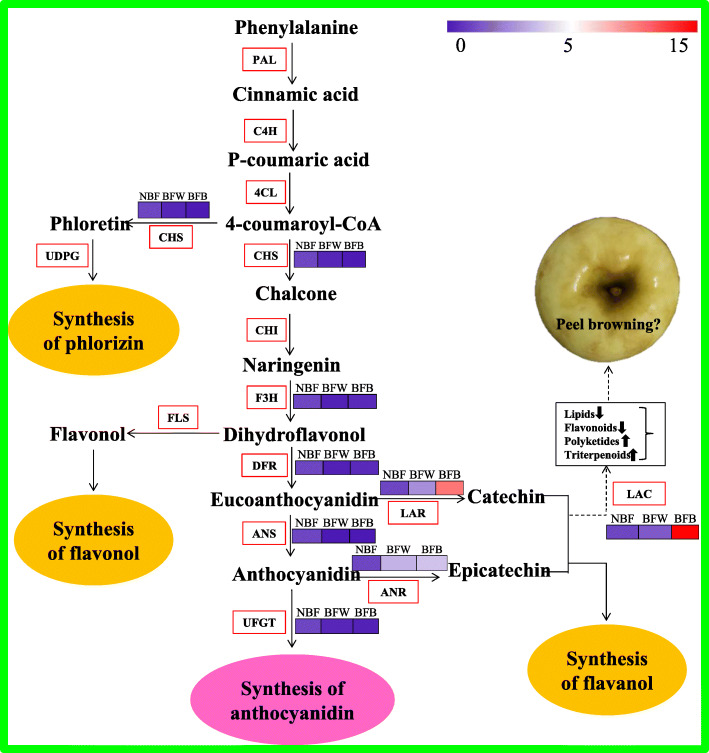
The metabolic pathway of flavonoids. Note: The color from purple to red means that gene expression increase successively. PAL: Phenylalanine ammonia-lyase; C4H: Cinnamate 4-hydroxylase; 4CL: 4-coumarate coenzyme A ligase; CHS: Chalone synthase; CHI: Chalcone isomerase; F3H: Flavanone 3-hydroxylase; DFR: Dihydroflavonol reductase; ANS: Anthocyanidin synthase; LAR: Leucoanthocyanidin reductase; ANR: Anthocyanidin reductase; UFGT: UGP glucose-flavonoid3-o- glucosyl; LAC: P-diphenol dioxygen oxidoreductase

It is well known, flavonoids are major secondary metabolites, including anthocyanin, flavone, flavonol, procyanidins, isoflavone, and orange ketone different subgroups [35], which play a series of roles in cells, inducing color changes and improving the antioxidant ability of the plant [36, 37]. Flavonoids are synthesized in plants through the phenylephrine metabolic pathway, starting from phenylalanine and catalyzed by the phenylalanine ammonialyase (*PAL*) to form cinnamic acid, then forms *p*-coumary-coa catalyzed by cinnimate 4-hydroxylase (*C4H*) and 4-coumarate coa ligase (*4CL*). Under the joint action of chalcone synthase (*CHS*), chalcone isomerase (*CHI*), flavanone 3-hydroxylase (*F3H*), and flavanone 3-hydroxylase (*DFR*), *p*-coumaniol-coa is transformed into flavonoid products [38]. The downstream structural gene *LAC* has been proved that its closely related to the oxidative polymerization of flavonoids on the phenylephrine metabolic pathway [39]. Previous studies have shown that laccase (*p*-diphenol: O_2_-oxidoreduacatse, E. C 1.10.3.2) encoded by *LAC* is directly related to the fruit browning [40]. Laccase as an intracellular enzyme, which is involved in the biosynthesis of lignin, capable of monolol oxidative polymerization, and participates in the polymerization of flavonoid substances [40]. The laccase encoded by *TT10* in the model plant *Arabidopsis*, as a flavonoid oxidase to catalyze oxidative polymerization of epicatechin to produce yellow and brown pigments different from colorless proanthocyanidins [41]. Epicatechin and procyanidin secreted into plasmids can be interpreted that in the process of seed dry cell death caused by broken vacuole, and epicatechin and former cyanine glycosides interact with *TT10*, originally colorless former cyanine glycosides and cell wall polysaccharides and other phenolic compounds form oxide compounds, can easily lead to organization dim [39]. Some laccase genes (such as *LAC14* and *LAC15*) also had a higher expression in the peel of fruit rust [42, 43]. In *litchi*, *ADE/LAC* is located in vacuoles and transported to the cell wall to function, and oxidative polymerization with catechin in peel occurs, accelerating degradation of procyanidins and promoting browning of peel [39]. It is interesting to note that laccases have a wider substrate range than PPO [44]. Similar to related PPO, laccases are capable of oxidizing not only *o*-diphenols, but also *p*-diphenols, methoxy-substituted monophenols, diamines and non-aromatics, despite the mechanism for many of these reactions has not been elucidated [45]. In our study, it was found that in the process of browning, *MdLAC14* was also expressed along with the high expression of *MdLAC7*, and we speculated that there was an interactive relationship between *MdLAC7* and *MdLAC14.* While, the content of catechin, epicatechin and proanthocyanidin were significantly decreased with browning, so, we believe that these compounds may be substrates for the new enzymatic browning induced by laccase. But, the specific molecular mechanism needs us to explore. Figure 13 shows the metabolic pathway of flavonoids.

In addition, through the analysis of metabolome data, we also found an interesting thing: after bagging, more triterpenes enriched in BFB, while the accumulation of triterpenes was not obvious in the peel of NBF or BFW. Terpenoids are widely found in nature and are the main components of some plant essences, resins, and pigments (such as rose oil, eucalyptus oil, turpentine) [46–48]. For instance, ganolucidic acid, first proposed by Kubota et al. [49], which is a highly oxidized lanolin triterpenoid, a triterpenoid commonly found in ganoderma lucidum. It is generally yellow-brown and has a very bitter taste [49]. So, we suspect that the excessive expression of triterpenes in the peel might be principally responsible for peel browning. Besides, the high expression of *MdLAC* was not only directly related to the decrease of flavonoids, but also related to the oxidative polymerization of terpenoids in fruits at maturity stage? The effect of bagging on the synthesis of triterpenes remains to be studied.

## Conclusions

In summary, the results of this study indicated that the destruction of cell structure, the increase of lipid peroxidation of cell membrane, the decrease of flavonoids and the increase of triterpenoids were the main reasons for the browning of peel about bagging ‘Rui Xue’. What’s more, whether a direct relationship between the high *MdLAC* expression, the decrease of flavonoids and the increase of triterpenes requires further investigations.

## Materials and methods

### Experimental design

The experiment was conducted at the Bai Shui County Apple Test Station of Northwest A & F University (35°2′ N, 109°6′ E) from May to October in 2019. The test station owns a warm temperate continental monsoon climate with an altitude of 915 m, annual mean rainfall of 578 mm, and an annual mean temperature of 11.4 °C. In the middle and late stage of fruit growth, the maximum temperature of atmosphere and inner bag in the daytime was 31.7 °C, the relative humidity was 40.7 and 35.9%, respectively, the minimum temperature at night was 4.1 °C and 4.6 °C, respectively, and the relative humidity was 100%, the details of the date are given in Supplemental Fig. S2. Scions of ‘Rui Xue’ were grafted to M26 roots stocks. 5-year-old fruit trees of ‘Rui Xue’ were used as materials (‘Rui Xue’ has passed the national cultivar certification in China. The trees and bud wood under rules of International Plant Variety Rights are available for research purposes, which can be obtained from Zhengyang Zhao at Northwest A&F University. The number of cultivar is CNA20151469.1). The trees were cultivated as spindle-shaped (1.5 × 3.0 m). Bagging was conducted in middle of May 2019. The external bag was brown and the inner was red (the specification of fruit bags was 155 mm × 180 mm, the outer paper was 57.0 g wood pulp composite paper, the outer color was pulp color, and the inner color was black, the inner paper was 32.0 g red paper coated with wax on both sides). Three rows of bagging and without bagging fruits were set as biological repeats, with ten plants in each line. The fruits were harvested with bags at the mature stage (October, 2019). The bagging fruits without browning (BFW) and bagging fruits with browning (BFB) were randomly selected as the experimental group, non-bagging fruits (NBF) were used as the control (CT). Thirty fruits with the same size, maturity, and without any mechanical damage were selected for analysis for each group. The fruit peels of the stalk cavity were sampled with a sterilized scalpel, and then immediately frozen in liquid nitrogen and stored at − 80 °C.

## Methods

### Classification of apple peel browning

According to the methods of Chen [17] (slightly modified), the bagged fruits were classified into five levels according to the browning area (expressed by S) of the peel (Fig. 1):

0, there was no brown spot on the surface of fruit;

I, the browning area was 0 < S ≤ 1/5;

II, the browning area was 1/5 < S ≤ 1/4;

III, the browning area was 1/4 < S ≤ 1/3;

IV, the browning area was 1/3 < S ≤ 1/2;

V, the browning area was 1/2 < S ≤ 1.

### Peel thickness

The thickness of the peel was determined by the paraffin section method [50]. Each peel section in every group was randomly selected at three different positions of the stalk cavity peel. The whole area of tissues was pictured under a 100-fold field of view, so as to ensure the consistent background light of each photo. We chose the outer three layers of cells as the critical for measuring the thickness of the peel. Image-pro Plus 6.0 (Media Cybernetics, Inc., Rockville, MD, USA) was used to measure the thickness (mm) of epidermis at five points of each slice with a 100-fold scale as the standard.

### Microstructure of the peel cells

The ultrastructure of cells was determined according to the method described by Chen [17]. The peel flakes were fixed on the glass-covered with 4% glutaraldehyde for 5 h and washed with ethanol with an increasing gradient (10–100%) for 10 min. Each piece of tissue was soaked in 100% ethanol for 10 min and then stored overnight in a dryer. The completely dehydrated tissues were transferred to the double-sided carbon-belted aluminum pile in a vacuum, then sputtered and gilded, and finely sliced. The visualized 3D model was analyzed at 20 kV using a scanning electron microscope (FEI Quanta 200, Thermo Fisher Scientific, Bedford, MA, USA).

### Metabolites profiling

An ACQUITY UPLC ultra-high-performance liquid phase series AB Triple TOF 5600 high-resolution mass spectrometer was used to determine liquid-mass coupling. For the determination, 80 mg samples were taken. Then, the internal standard (20 μL) (L-2-chlorophenylalanine, 0.3 mg/mL; Lyso PC17:0; 0.01 mg/mL, all solutions were configured with methanol) and 1.0 mL methanol:water (V:V = 7:3, contains 0.01 mol/L BHT (2,6-di-tert-butyl-p-cresol)) were mixed. Then, two small steel balls were added, and the sample pre-cooled at − 20 °C for 2 min before being placed in the grinding machine (60 Hz, 2 min). After 30 min of ultrasonic extraction, the sample was left to stand at − 20 °C for 20 min, then centrifuged for 10 min (13,000 rpm, 4 °C). The supernatant (300 μL) and shake it dry, then use 400 μL methanol:water (V:V = 1:4) to redissolve it, vortex for 30 s, and ultrasound for 2 min. After centrifugation for 10 min (13,000 rpm, 4 °C), using a syringe to absorb 150 μL of the superfluid, and using 0.22 μL of the organic phase pinhole filter to filter it. Then, the filtered organic phase was transferred to the LC injection vial and stored at − 80 °C until LC-MS analysis. Three biological repeats were tested in each group. The quality control sample (QC) was prepared by mixing an equal volume of the extract of all samples; the final volume of each QC was the same as the sample volumes. All extracted reagents were pre-cooled at − 20 °C before use.

### Membrane lipid peroxidation

MDA was measured according to the method described by Sun [51] (with minor modifications). We took 1.0 mL of the sample supernatant extracted above and added 2.0 mL 0.67% thiobarbituric acid (TBA). Samples containing only 1.0 mL water were set as a negative control. Next, the samples were placed in a boiling water bath for 15 min, and then rapidly cooled by immersion in cold water, and poured them into 10 mL centrifuge tubes. All tubes were centrifuged at 400 rpm for 20 min and determining the absorbance of all samples at 600 nm, 532 nm, and 450 nm used a spectrophotometer.

O_2_^−^ was measured according to the method described by Zhao [52] (with minor modifications). Two point zero grams fresh samples were weighed, then, using 65 mmol·L^− 1^ phosphate buffer (pH 7.8) to extract, constant volume to 10 ml, centrifuged (10,000 rpm, 10 min), and the supernatant was extracted for later use. Extracting 2.0 ml of supernatant, then, adding 1.5 ml of phosphate buffer and 0.5 ml of hydroxylamine hydrochloride, mixing and keeping warm in water bath at 25 °C for 20 min. Extracting 2.0 ml supernatant after bathed, adding 2.0 ml 7 mmol·L^− 1^ α-naphthylamine and 2.0 ml 17 mmol·L^− 1^ sulfanilic acid, bathing at 30 °C for 30 min, measuring the absorbance of all samples at 530 nm used a spectrophotometer.

The content of H_2_O_2_ was measured according to the method described by Zhao [52]. Two point zero grams of fresh plant tissue was weighed, adding 2.0 ml of pre-cooled acetone. Then, centrifugation at 3000 rpm for 10 min. Then, extracting 0.3 ml supernatant, adding 0.1 ml of 5% titanium sulfate and 0.2 ml of concentrated ammonia, centrifuged (3000 rpm 10 min), then, the precipitation with acetone repeated washing 3 ~ 5 times, adding 5.0 ml sulfuric acid (2.0 mmol·L^− 1^), after being completely dissolved, measuring the absorbance of all samples at 415 nm used a spectrophotometer.

Cell membrane permeability was measured according to a simplified method [52]. Briefly, fruits were cleand with tap water, and then rinsed twice with distilled water. Zero point five grams peel of stalk cavity was taken into 50 ml centrifugal tube, 20 ml of distilled water added, shaking. Then the conductivity was measured using DDS-307 conductivity meter (Shanghai Precision Scientific Instrument Co. Ltd., Shanghai, China). The nozzle seals were put in a boiling water bath for 10 min, cooled by tap water, shaken and conductance measured according to the formula to calculate the cell membrane permeability.

### The activities of SOD, CAT and POD

The activities of SOD, CAT and POD were measured according to the method described by Zhao [52]. Briefly, 0.5 g fresh sample was weighed, adding 4.0 ml phosphate buffer (0.05 mol·L^− 1^ pH 7.8) to extract, then, centrifuged (4 °C 3000 rpm 10 min), all extracted reagents were pre-cooled at − 20 °C before use. The SOD activity was measured by Nitrogen-Blue Tetrazole photochemical reduction method. The CAT activity using the method of Kar and Mishra [45, 53]. The POD activity was determined by Guaiacol method.

### Total antioxidant capacity

According to the methods of Lee and Wicker [54], 1.0 g peel was grounded in liquid nitrogen. Then, a 1.5 ml alcohol-acetone mixture (v:v = 7:3) was added and incubated at 37 °C for 1 h. The homogenate was then centrifuged at 13,000 rpm at 20 °C for 10 min. The supernatant was immediately stored in the − 20 °C refrigerator, which used to determine the total antioxidant activity and content of antioxidant substances.

Total phenol content was spectrophotometrically determined according to the Folin-Ciocalteu method [55]. Flavonoids were determined by Nitrite-Aluminum Chloride [56], using rutin as standard, and the results were calculated using the rutin calibration curve. Flavanol content was detected with 4-DMACA [39]. The total antioxidant capacity of peel was determined by Iron reduction ability method.

### Phenolic acid composition

According to the method described by Zhang [57] to measure the phenolic acid composition (with minor modifications). Briefly, 0.1 g fresh sample was weighed, and place it in a 1.5 mL centrifuge tube, then add 1.0 mL of extract (Methanol: Water: Formic acid = 25:24:1; v:v:v), ultrasonic for 20 min at 25 °C (40 Hz, 100 W), then shocked for 20 min, centrifuged (10,000 rpm 15 min). After, the supernatant was absorbed with a syringe, it was filtered into a brown amber bottle with 0.25 μm organic nylon filter, using Liquid Mass Spectrometry to determinate phenolic acid components. Mobile liquid A was 0.1% formic acid water, and mobile liquid B was 100% methanol.

### Total RNA extraction and qPCR analysis

Total RNA was extracted from the apple of peel (set up three independent replicates), using the RNA Plant Plus Reagent Kit (TIANGEN, China) and reverse transcribed into cDNA using PrimeScript™RT Reagent Kit (Takara, Dalian, China). Quantitative polymerase chain reactions (qPCRs) were carried out in the ABI7500 System using SYBR Premix Ex Taq (Takara). The calculation method for qRT-PCR was 2^−△△CT^. At least three replicates for each sample were used for qPCR. The primers used for qPCR are listed in Table 1.

**Table 1:** The primer sequences for real-time PCR

Gene name	Gene ID	Forward primer sequence	Reverse primer sequence
*MdActin*	MD12G1140800	TGACCGAATGAGCAAGGAAATTACT	TACTCAGCTTTGGCAATCCACATC
*MdDFR*	MD15G1024100	AGGAACTGTGAATGTGGAGGAGC	AGGAACTGTGAATGTGGAGGAGC
*MdANS*	MD06G1071600	TCCCAAAAGAGTACATCAGACCTAA	ACACCCCAGTCCACAGTTGC
*MdLAR*	MD16G1048500	TCTTGGCCCTACTTTGACAAC	AGAGTTCCCACTTCCACATCA
*MdANR*	MD10G1311100	CCACCTCACAGCACTACAAGAG	GCAAATTTCCAAGCTGTCTTCT
*MdUFGT*	MD01G1234400	GCAAGCCGCAGGAGGACATA	CGGAGATCCAGAAAGTGACCCA
*MdLAC7*	MD04G1142300	ATGGCACGGTTAGCCTTT	CTAACATTTCGGCAGATCAAGT
*MdLAC12*	MD01G1159400	TGGAGGCACTCAACGCATTT	GATCTATAGATTCTTAACAA
*MdLAC14*	MD02G1264400	ATGGCCTCAGACAAGAAAATAC	CTAAGACTTGGAACAAGGAGGCA
*MdLAC15*	MD07G1308000	ATGAGTATTTTAATTAGCTTGC	ACATGGAGGCATTCCTTGTGG
*MdLAC16*	MD07G1307400	ATGCATCTCCACGGATTCAG	TGTTAACATGGAGGCATTCC

### Statistical analysis

Three biological repeats were taken from the experimental data. Microsoft Excel 2010, SigmaPlot 13, and Progenesis QI V2.3 software were used to process and plot the data, and SPSS 24.0 software was used to conduct a one-way analysis of variance (ANOVA). The data were analyzed using the t-test. The data were expressed as mean ± standard error. Significance was defined at *p* < 0.05.

## Supplementary Information

**Additional file 1: Figure S1.** Hierarchical clustering graph for screening different metabolites. A: BFW-NBF group; B: BFW-BFB group. Note: The abscissa represents the sample name, and the ordinate represents the differential metabolites. The color from green to red indicates the expression abundance of metabolites from low to high, that is, the redder indicates the expression abundance of different metabolites.

**Additional file 2: Figure S2.** The changing of temperature and humidity in the early stage of harvest. Note: The abscissa represents the date, the primary ordinate axis represents the daily highest temperature, the secondary ordinate axis represents the average relative humidity. AHtemp represents the daily highest temperature of NBF; BHtemp represents the daily highest temperature of BF; Ahumi represents the average relative humidity of NBF; Bhumi represents the average relative humidity of BF.

**Additional file 3: Table S1.** Detailed metabolites of top 50 about BFW-NBF group.

**Additional file 4: Table S2.** Detailed metabolites of top 50 about BFW-BFB group.

## Data Availability

The raw metabonomics data have been deposited to the EMBL-EBI MetaboLights database with the identifier MTBLS2384. The complete dataset can be accessed here: https://www.ebi.ac.uk/metabolights/MTBLS2384/descriptors.

## Abbreviations

BFW: Bagging fruits without browning
BFB: Bagging fruits with browning
NBF: Non-bagging fruits
PLS-DA: Partial least squares discriminant analysis
VIP: Variable importance of projection
MDA: Malondialdehyde
H_2_O_2_: Hydrogen peroxide
O_2_^−^: Superoxide anion
FAO: Food and Agriculture Organization of the United Nations
TCA: Tricarboxylic acid cycle
GABA: γ-aminobutyric acid
QC: Quality control
PCA: Principal component analysis
KEGG: Kyoto encyclopedia of genes and genomes
ROS: Reactive oxygen species
SOD: Superoxide dismutase
CAT: Catalase from micrococcus lysodeiktic
POD: Peroxidase
TP: Total phenols
TFA: Total flavanols
TFO: Total flavonoids
PPO: Enzyme polyphenol oxidase
PAL: Phenylalanine ammonia-lyase
C4H: Cinnamate 4-hydroxylase
4CL: 4-coumarate coenzyme A ligase
CHS: Chalone synthase
CHI: Chalcone isomerase
F3H: Flavanone 3-hydroxylase
DFR: Dihydroflavonol reductase
ANS: Anthocyanidin synthase
LAR: Leucoanthocyanidin reductase
ANR: Anthocyanidin reductase
UFGT: UGP glucose-flavonoid3-o-glucosyl
LAC: P-diphenol dioxygen oxidoreductase
qRT-PCR: Quantitative real-time PCR
cDNA: Complementary DNA
4-DMACA: 4-(Dimethylamino) cinnamaldehyde
