# Supplementary figures and images for: Metabolomic insights into the browning of the peel of bagging ‘Rui Xue’ apple fruit
Source: BMC Plant Biol. 2021 May 8;21:209. doi: 10.1186/s12870-021-02974-y (PMC8106160; doi:10.1186/s12870-021-02974-y)

**Figure S1.**


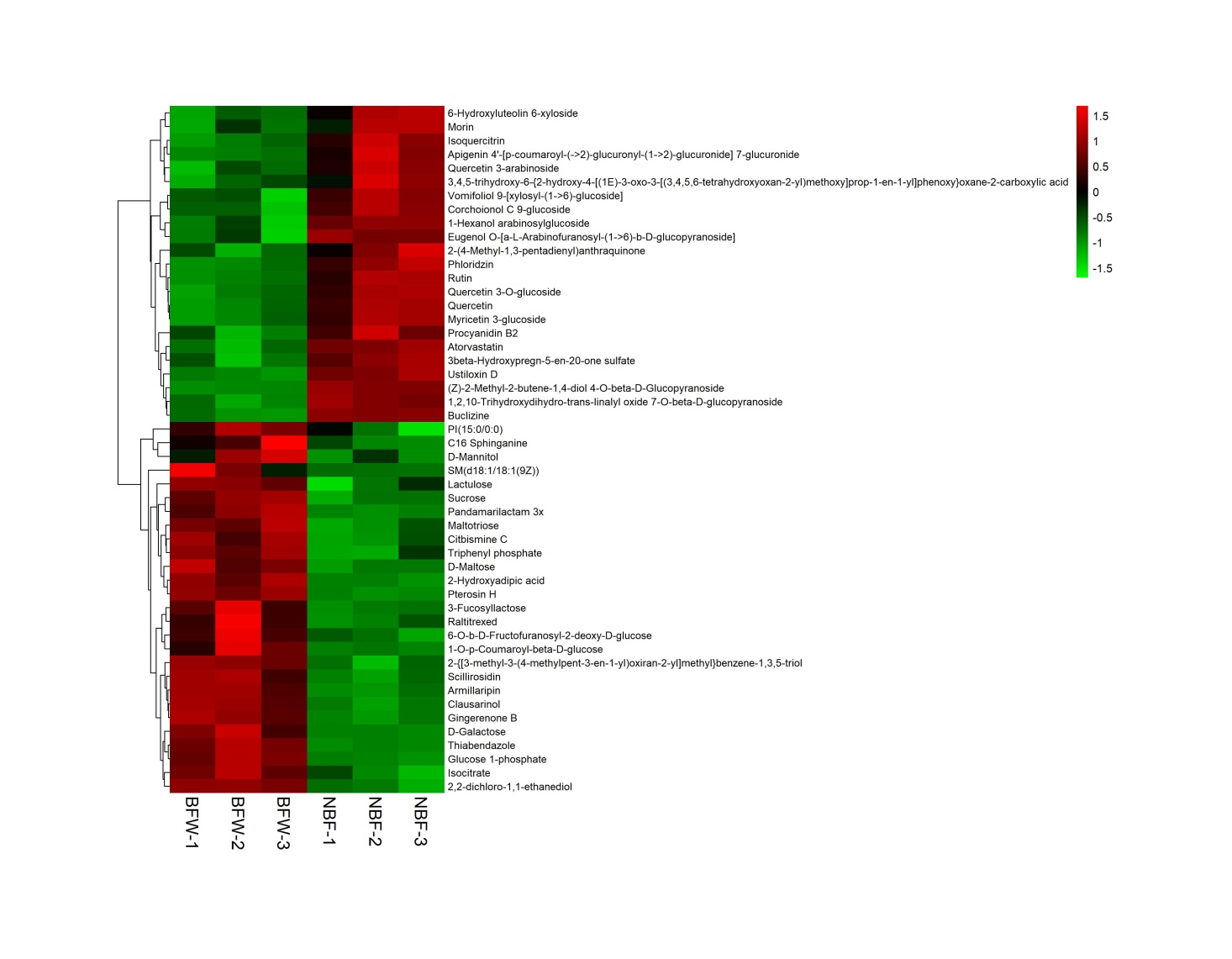


A


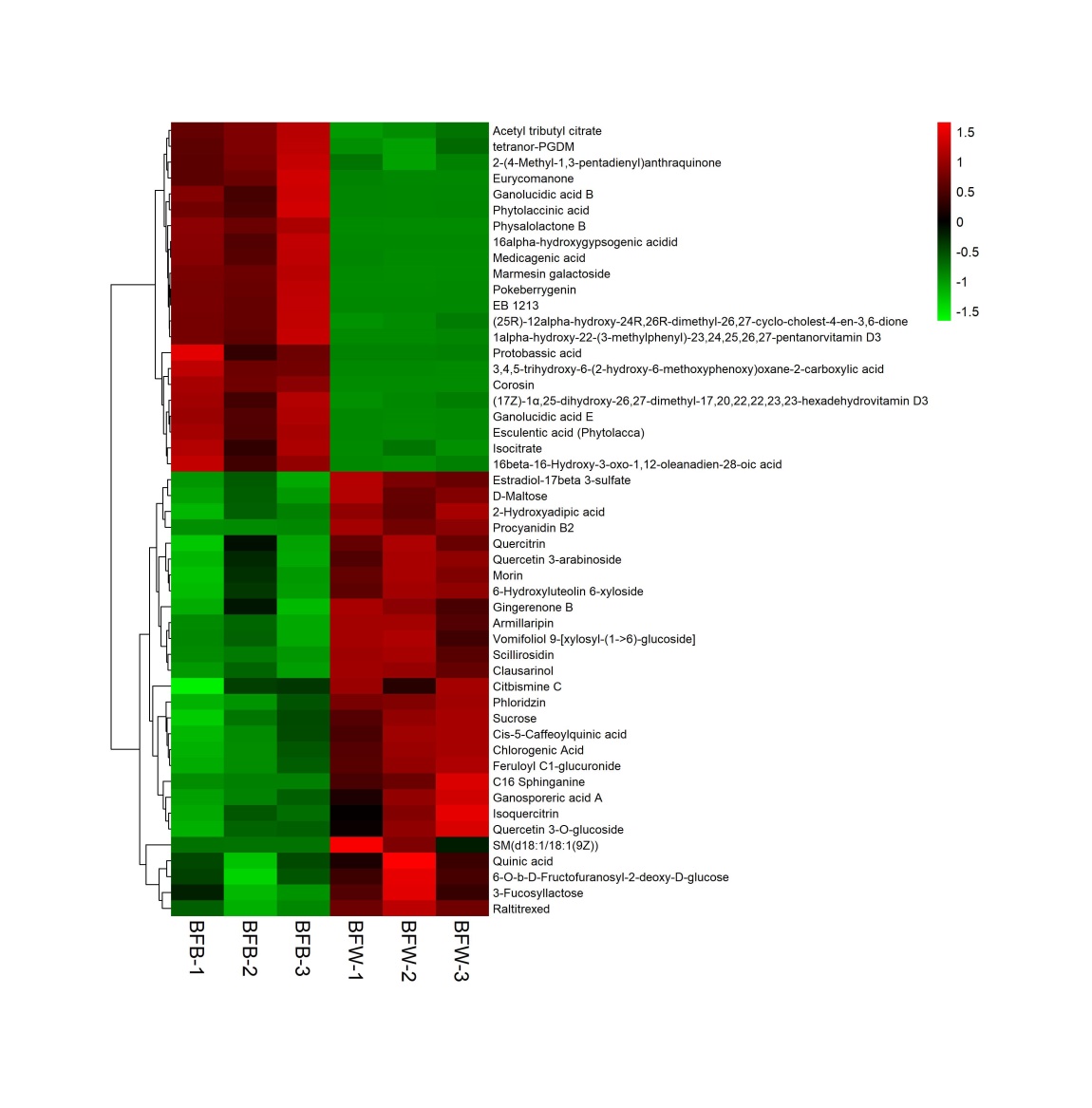


B

B

Supplement: Supplementary file 1 — Additional file 1: Figure S1. Hierarchical clustering graph for screening different metabolites. A: BFW-NBF group; B: BFW-BFB group. Note: The abscissa represents the sample name, and the ordinate represents the differential metabolites. The color from green to red indicates the expression abundance of metabolites from low to high, that is, the redder indicates the expression abundance of different metabolites. [file 12870_2021_2974_MOESM1_ESM.docx]

**Figure S2.**


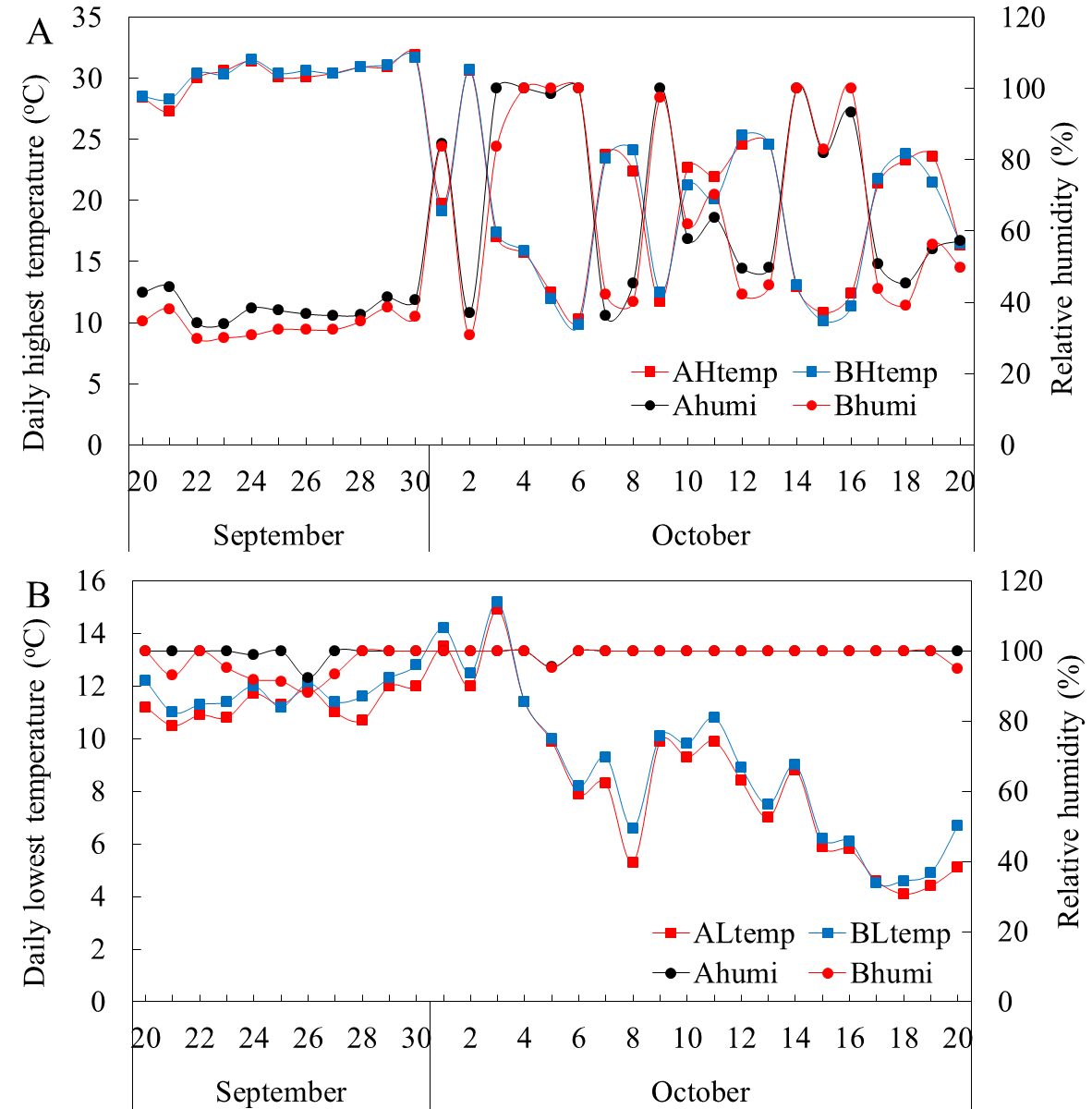

Supplement: Supplementary file 2 — Additional file 2: Figure S2. The changing of temperature and humidity in the early stage of harvest. Note: The abscissa represents the date, the primary ordinate axis represents the daily highest temperature, the secondary ordinate axis represents the average relative humidity. AHtemp represents the daily highest temperature of NBF; BHtemp represents the daily highest temperature of BF; Ahumi represents the average relative humidity of NBF; Bhumi represents the average relative humidity of BF. [file 12870_2021_2974_MOESM2_ESM.docx]
