# Supplementary material for: Metabolomic insights into the browning of the peel of bagging ‘Rui Xue’ apple fruit
Source: BMC Plant Biol. 2021 May 8;21:209. doi: 10.1186/s12870-021-02974-y (PMC8106160; doi:10.1186/s12870-021-02974-y)
Supplement: Supplementary file 3 — Additional file 3: Table S1. Detailed metabolites of top 50 about BFW-NBF group. [file 12870_2021_2974_MOESM3_ESM.docx]

**Table S1** **Detailed metabolites of top 50 about BFW-NBF group.**

| **Classification** | **Metabolites** | **log2(FC)** | **Formula** | **VIP** | **P-value** | **FC** | **average(BFW)** | **average(NBF)** |
| --- | --- | --- | --- | --- | --- | --- | --- | --- |
| Flavonoids and flavonoids compounds | Apigenin | -3.36 | C42H40O25 | 3.96 | 0.0114 | 0.0977 | 102.89 | 1053.27 |
|  | Rutin | -2.52 | C27H30O16 | 5.41 | 0.0046 | 0.1752 | 369.79 | 2110.50 |
|  | Myricetin 3-glucoside | -1.73 | C21H20O13 | 5.47 | 0.0038 | 0.3018 | 766.42 | 2539.51 |
|  | Isoquercitrin | -1.56 | C21H20O12 | 18.18 | 0.0073 | 0.3383 | 10129.93 | 29945.85 |
|  | Quercetin | -1.54 | C15H10O7 | 6.95 | 0.0031 | 0.3442 | 1499.79 | 4357.91 |
|  | Quercetin 3-O-glucoside | -1.46 | C21H20O12 | 13.27 | 0.0042 | 0.3624 | 5974.78 | 16488.13 |
|  | Phloridzin | -0.87 | C21H24O10 | 5.75 | 0.0036 | 0.5474 | 2392.84 | 4371.24 |
|  | 6-Hydroxyluteolin 6-xyloside | -0.59 | C20H18O11 | 7.52 | 0.0159 | 0.6635 | 6966.43 | 10499.56 |
|  | Procyanidin B2 | -0.50 | C30H26O12 | 3.47 | 0.0090 | 0.7065 | 1755.65 | 2485.05 |
|  | Quercetin 3-arabinoside | -0.49 | C20H18O11 | 10.28 | 0.0151 | 0.7111 | 16163.97 | 22730.88 |
|  | Morin | -0.33 | C15H10O7 | 5.95 | 0.0477 | 0.7937 | 9227.06 | 11624.65 |
|  | Clausarinol | 1.23 | C24H30O6 | 9.40 | 0.0007 | 2.3521 | 9016.74 | 3833.49 |
|  | Gingerenone B | 1.54 | C22H26O6 | 8.98 | 0.0005 | 2.9125 | 7223.55 | 2480.21 |
|  |  |  |  |  |  |  |  |  |
| Lipid compound | D-Glucopyranoside | -4.09 | C11H20O7 | 3.24 | 0.0000 | 0.0586 | 38.29 | 653.11 |
|  | Hydroxypregn sulfate | -1.13 | C21H32O5S | 3.45 | 0.0031 | 0.4565 | 598.61 | 1311.35 |
|  | 1-Hexanol arabinosylglucoside | -0.68 | C17H32O10 | 5.28 | 0.0037 | 0.6251 | 2849.32 | 4558.17 |
|  | Corchoionol C 9-glucoside | -0.52 | C19H30O8 | 5.10 | 0.0053 | 0.6982 | 3653.17 | 5232.05 |
|  | Vomifoliol | -0.41 | C24H38O12 | 5.88 | 0.0108 | 0.7520 | 6542.67 | 8700.63 |
|  | 2-Hydroxyadipic acid | 0.62 | C6H10O5 | 6.05 | 0.0004 | 1.5386 | 6239.77 | 4055.55 |
|  | SM(d18:1/18:1(9Z)) | 5.28 | C41H81N2O6P | 9.67 | 0.0483 | 38.8031 | 6757.47 | 174.15 |
|  | Scillirosidin | 0.95 | C26H34O6 | 9.42 | 0.0024 | 1.9345 | 10968.92 | 5670.15 |
|  | PI(15:0/0:0) | 0.68 | C24H47O12P | 3.35 | 0.0341 | 1.5979 | 1991.77 | 1246.48 |
|  | C16 Sphinganine | 0.90 | C16H35NO2 | 4.73 | 0.0374 | 1.8610 | 3361.63 | 1806.35 |
|  |  |  |  |  |  |  |  |  |
| Organic acids and their derivatives | Oxane-2-carboxylic acid | -0.46 | C21H26O15 | 3.25 | 0.0387 | 0.7246 | 1815.43 | 2505.52 |
|  | Raltitrexed | 0.24 | C21H22N4O6S | 4.57 | 0.0216 | 1.1831 | 8824.75 | 7459.10 |
|  | Isocitrate | 0.65 | C6H8O7 | 5.16 | 0.0036 | 1.5683 | 4403.58 | 2807.95 |
|  | Ustiloxin D | -1.63 | C23H34N4O8 | 3.44 | 0.0001 | 0.3225 | 330.92 | 1026.14 |
|  | Coumaroy D-glucose | 0.74 | C15H18O8 | 3.27 | 0.0109 | 1.6647 | 1698.15 | 1020.12 |
|  |  |  |  |  |  |  |  |  |
|  | Lactulose | 0.23 | C12H22O11 | 8.12 | 0.0088 | 1.1734 | 28830.22 | 24570.23 |
|  | Glucose 1-phosphate | 0.47 | C6H13O9P | 3.27 | 0.0004 | 1.3895 | 2243.80 | 1614.86 |
|  | D-Mannitol | 0.25 | C6H14O6 | 3.24 | 0.0484 | 1.1883 | 4904.57 | 4127.25 |
|  | Sucrose | 0.29 | C12H22O11 | 12.74 | 0.0007 | 1.2267 | 53090.85 | 43278.80 |
|  | D-Galactose | 0.36 | C6H12O6 | 4.03 | 0.0024 | 1.2856 | 4380.39 | 3407.34 |
|  | D-Maltose | 0.45 | C12H22O11 | 4.75 | 0.0016 | 1.3674 | 5112.80 | 3739.19 |
|  | Fructofuranosy | 0.46 | C12H22O10 | 5.85 | 0.0157 | 1.3773 | 7973.82 | 5789.45 |
|  | 3-Fucosyllactose | 0.47 | C18H32O15 | 12.41 | 0.0087 | 1.3842 | 34709.81 | 25075.87 |
|  | Maltotriose | 0.68 | C18H32O16 | 3.60 | 0.0023 | 1.5982 | 2129.93 | 1332.71 |
| Carbohydrate | D-glucopyranoside | -0.81 | C16H30O10 | 7.97 | 0.0002 | 0.5686 | 4951.46 | 8708.86 |
|  |  |  |  |  |  |  |  |  |
| Benzene ring compound | Eugenol | -0.41 | C21H30O11 | 3.24 | 0.0045 | 0.7534 | 2020.58 | 2681.90 |
|  | Anthraquinone | -0.39 | C20H16O2 | 5.26 | 0.0246 | 0.7654 | 5818.93 | 7602.48 |
|  | Benzene-1,3,5-triol | 0.25 | C16H22O4 | 3.60 | 0.0010 | 1.1867 | 4827.81 | 4068.19 |
|  | Triphenyl phosphate | 0.31 | C18H15O4P | 3.62 | 0.0040 | 1.2387 | 4198.10 | 3389.10 |
|  | Thiabendazole | 0.43 | C10H7N3S | 3.81 | 0.0003 | 1.3456 | 3339.24 | 2481.65 |
|  | Pandamarilactam 3x | 0.31 | C13H17NO3 | 3.65 | 0.0011 | 1.2379 | 4146.70 | 3349.89 |
|  | Citbismine C | 0.36 | C37H36N2O11 | 13.76 | 0.0031 | 1.2856 | 52618.61 | 40927.83 |
|  | Pterosin H | 0.70 | C15H19ClO | 5.09 | 0.0001 | 1.6205 | 3969.86 | 2449.78 |
|  | Buclizine | -1.39 | C28H33ClN2 | 4.40 | 0.0001 | 0.3810 | 698.88 | 1834.50 |
|  |  |  |  |  |  |  |  |  |
| Sesquiterpenoids | Armillaripin | 1.02 | C24H30O6 | 8.23 | 0.0009 | 2.0367 | 7866.30 | 3862.31 |
|  |  |  |  |  |  |  |  |  |
| Others | 2,2-dichloro-1,1-ethanediol | 0.42 | C2H4Cl2O2 | 4.77 | 0.0002 | 1.3345 | 5333.18 | 3996.24 |
|  | Atorvastatin | -1.66 | C33H35FN2O5 | 3.80 | 0.0010 | 0.3169 | 396.24 | 1250.39 |
