# Supplementary material for: Metabolomic insights into the browning of the peel of bagging ‘Rui Xue’ apple fruit
Source: BMC Plant Biol. 2021 May 8;21:209. doi: 10.1186/s12870-021-02974-y (PMC8106160; doi:10.1186/s12870-021-02974-y)
Supplement: Supplementary file 4 — Additional file 4: Table S2. Detailed metabolites of top 50 about BFW-BFB group. [file 12870_2021_2974_MOESM4_ESM.docx]

**Table S2 Detailed metabolites of top 50 about BFW-BFB group.**

| **Classification** | **Metabolites** | **log2(FC)** | **Formula** | **VIP** | **P-value** | **FC** | **average(BFB)** | **average(BFW)** |
| --- | --- | --- | --- | --- | --- | --- | --- | --- |
| Flavonoids and flavonoids compounds | Procyanidin B2 | -5.77 | C30H26O12 | 3.65 | 0.0001 | 0.0183 | 32.13 | 1755.65 |
|  | Phloridzin | -2.36 | C21H24O10 | 3.84 | 0.0009 | 0.1943 | 465.03 | 2392.84 |
|  | Hydroxyluteolin | -1.25 | C20H18O11 | 5.49 | 0.0039 | 0.4208 | 2931.32 | 6966.43 |
|  | Quercetin 3-arabinoside | -1.14 | C20H18O11 | 8.04 | 0.0076 | 0.4539 | 7337.58 | 16163.97 |
|  | Gingerenone B | -1.11 | C22H26O6 | 5.25 | 0.0131 | 0.4640 | 3351.53 | 7223.55 |
|  | Quercetin 3-O-glucoside | -0.98 | C21H20O12 | 4.53 | 0.0194 | 0.5070 | 3029.24 | 5974.78 |
|  | Isoquercitrin | -0.92 | C21H20O12 | 5.71 | 0.0242 | 0.5281 | 5350.01 | 10129.93 |
|  | Quercitrin | -0.80 | C21H20O11 | 3.94 | 0.0141 | 0.5763 | 2954.61 | 5126.66 |
|  | Morin | -1.07 | C15H10O7 | 5.98 | 0.0054 | 0.4779 | 4410.02 | 9227.06 |
|  | Clausarinol | -2.33 | C24H30O6 | 7.46 | 0.0005 | 0.1995 | 1798.42 | 9016.74 |
|  | Eurycomanone | 5.29 | C20H24O9 | 3.59 | 0.0018 | 39.1320 | 1767.85 | 45.18 |
|  | Marmesin galactoside | 7.94 | C20H24O9 | 3.74 | 0.0003 | 246.0528 | 1832.26 | 7.45 |
|  |  |  |  |  |  |  |  |  |
| Lipid compound | SM(d18:1/18:1(9Z)) | -7.70 | C41H81N2O6P | 6.59 | 0.0454 | 0.0048 | 32.51 | 6757.47 |
|  | C16 Sphinganine | -2.88 | C16H35NO2 | 4.63 | 0.0040 | 0.1354 | 455.15 | 3361.63 |
|  | Scillirosidin | -1.68 | C26H34O6 | 7.63 | 0.0005 | 0.3121 | 3423.02 | 10968.92 |
|  | Estradiol-17beta 3-sulfate | -1.27 | C18H24O5S | 4.79 | 0.0011 | 0.4145 | 2140.14 | 5163.78 |
|  | Vomifoliol | -0.75 | C24H38O12 | 4.46 | 0.0029 | 0.5956 | 3897.13 | 6542.67 |
|  | 2-Hydroxyadipic acid | -0.74 | C6H10O5 | 4.36 | 0.0011 | 0.5993 | 3739.47 | 6239.77 |
|  | tetranor-PGDM | 0.47 | C16H24O7 | 5.36 | 0.0010 | 1.3852 | 13607.19 | 9823.28 |
|  | Cyclo-cholest | 4.44 | C29H44O4 | 5.82 | 0.0007 | 21.6630 | 4656.65 | 214.96 |
|  | Physalolactone B | 5.94 | C30H44O6 | 3.67 | 0.0001 | 61.4751 | 1772.63 | 28.83 |
|  | EB 1213 | 6.65 | C31H44O4 | 7.43 | 0.0006 | 100.3184 | 7302.86 | 72.80 |
|  | Pentanorcholecalciferol | 6.99 | C29H40O2 | 3.61 | 0.0010 | 127.3193 | 1735.74 | 13.63 |
|  |  |  |  |  |  |  |  |  |
| Organic acids and their derivatives | Feruloyl C1-glucuronide | -2.38 | C16H18O10 | 4.16 | 0.0013 | 0.1925 | 540.44 | 2807.72 |
|  | Chlorogenic Acid | -2.03 | C16H18O9 | 6.05 | 0.0017 | 0.2452 | 1562.80 | 6372.80 |
|  | Cis-5-Caffeoylquinic acid | -1.72 | C16H18O9 | 3.57 | 0.0036 | 0.3044 | 741.48 | 2436.17 |
|  | Raltitrexed | -0.84 | C21H22N4O6S | 5.44 | 0.0013 | 0.5590 | 4933.41 | 8824.75 |
|  | Quinic acid | -0.51 | C7H12O6 | 5.23 | 0.0497 | 0.7040 | 10037.97 | 14259.24 |
|  | Acetyl tributyl citrate | 0.52 | C20H34O8 | 11.13 | 0.0006 | 1.4374 | 53416.67 | 37162.64 |
|  | Isocitrate | 1.19 | C6H8O7 | 6.50 | 0.0035 | 2.2883 | 10076.85 | 4403.58 |
|  |  |  |  |  |  |  |  |  |
| Benzene ring compound | Citbismine C | -0.25 | C37H36N2O11 | 7.46 | 0.0302 | 0.8422 | 44315.72 | 52618.61 |
|  | Anthraquinone | 0.84 | C20H16O2 | 5.90 | 0.0013 | 1.7936 | 10436.71 | 5818.93 |
|  |  |  |  |  |  |  |  |  |
| Carbohydrate | D-Maltose | -0.73 | C12H22O11 | 3.93 | 0.0010 | 0.6029 | 3082.58 | 5112.80 |
|  | D-Fructofuranosyl | -0.47 | C12H22O10 | 3.91 | 0.0277 | 0.7198 | 5739.22 | 7973.82 |
|  | 3-Fucosyllactose | -0.44 | C18H32O15 | 7.88 | 0.0256 | 0.7376 | 25602.66 | 34709.81 |
|  | Sucrose | -0.25 | C12H22O11 | 7.88 | 0.0044 | 0.8434 | 44774.77 | 53090.85 |
|  |  |  |  |  |  |  |  |  |
| Tritererpenoids | Ganosporeric acid A | -2.09 | C30H38O8 | 3.96 | 0.0099 | 0.2353 | 670.37 | 2848.42 |
|  | Medicagenic acid | 3.76 | C30H46O6 | 5.02 | 0.0007 | 13.5111 | 3568.21 | 264.09 |
|  | 12-oleanadien-28-oic acid | 4.57 | C30H44O4 | 3.65 | 0.0021 | 23.7357 | 1833.38 | 77.24 |
|  | Esculentic acid | 4.79 | C30H46O6 | 12.38 | 0.0006 | 27.6745 | 20727.80 | 748.99 |
|  | Oxane-2-carboxylic acid | 5.06 | C13H16O9 | 6.64 | 0.0004 | 33.3201 | 5887.41 | 176.69 |
|  | Phytolaccinic acid | 5.27 | C31H48O6 | 9.98 | 0.0025 | 38.4988 | 13724.52 | 356.49 |
|  | Ganolucidic acid B | 5.62 | C30H46O6 | 12.35 | 0.0023 | 49.2307 | 20802.54 | 422.55 |
|  | Protobassic acid | 6.11 | C30H48O6 | 5.10 | 0.0077 | 68.9545 | 3599.93 | 52.21 |
|  | Ganolucidic acid E | 6.25 | C30H44O5 | 21.84 | 0.0006 | 76.1138 | 63065.36 | 828.57 |
|  | Hydroxygypsogenic acidid | 6.55 | C30H46O6 | 29.67 | 0.0010 | 93.5205 | 117180.35 | 1252.99 |
|  | Pokeberrygenin | 6.99 | C31H48O6 | 17.99 | 0.0005 | 127.1633 | 42655.92 | 335.44 |
|  | Corosin | 9.44 | C30H46O7 | 5.11 | 0.0001 | 693.0599 | 3361.47 | 4.85 |
|  |  |  |  |  |  |  |  |  |
| Sesquiterpenoids | Armillaripin | -1.10 | C24H30O6 | 5.65 | 0.0012 | 0.4680 | 3681.11 | 7866.30 |
|  |  |  |  |  |  |  |  |  |
| Others | Hexadehydrovitamin D3 | 3.53 | C29H42O3 | 6.39 | 0.0015 | 11.5557 | 5894.20 | 510.07 |
